# Certified reference materials for testing of the presence/absence of *Staphylococcus aureus* enterotoxin A (SEA) in cheese

**DOI:** 10.1007/s00216-016-9642-5

**Published:** 2016-05-24

**Authors:** Reinhard Zeleny, Yacine Nia, Heinz Schimmel, Isabelle Mutel, Jacques-Antoine Hennekinne, Håkan Emteborg, Jean Charoud-Got, Frédéric Auvray

**Affiliations:** European Commission, Joint Research Centre, Institute for Reference Materials and Measurements (IRMM), Retieseweg 111, 2440 Geel, Belgium; Laboratory for Food Safety, Université Paris-Est, ANSES, 94700 Maisons-Alfort, France

**Keywords:** Staphylococcal enterotoxins, SEA, Presence/absence testing, Certified reference material, Recovery study

## Abstract

*Staphylococcal* enterotoxins (SEs) account for a substantial number of food-poisoning outbreaks. European legislation (Commission Regulation 1441/2007) stipulates the reference procedure for SE analysis in milk and dairy products, which is based on extraction, dialysis concentration and immunochemical detection using one of two approved assays (VIDAS^®^ SET2, Ridascreen^®^ SET Total). However, certified reference materials (CRMs) are lacking to support laboratories in performing reliable detection of *Staphylococcus aureus* enterotoxin A (SEA) in relevant matrices at sub-nanogram per gram levels. The certification of a set of three reference materials (blank and two SEA-containing materials) for testing of the presence/absence of SEA in cheese is described. The reference procedure was applied in an intercomparison with 15 laboratories, and results were reported in a qualitative manner (presence or absence of SEA in the sample). No false-negative or false-positive results were obtained. The certified values were stated as diagnostic specificity (blank material) or diagnostic sensitivity (SEA-containing materials) and were 100 % in all cases. Stability studies demonstrated suitable material stability when stored cooled or frozen. An in-house study on the recovery of SEA in the cheese materials using a double-sandwich enzyme-linked immunosorbent assay (ELISA) revealed comparable recovery values of around 45 % at the two spiking levels and in both the SEA-containing CRMs as well as blank CRM freshly spiked prior to analysis. The values were also comparable over time and among different analysts. The materials provide valuable support to laboratories for method validation and method performance verification and will increase the reliability of measuring SEA in cheese.

**Electronic supplementary material:**

The online version of this article (doi:10.1007/s00216-016-9642-5) contains supplementary material, which is available to authorized users.

## Introduction

Staphylococcal enterotoxins (SEs) are released into food by certain strains of coagulase-positive Staphylococci (CPS), typically *Staphylococcus aureus*. These proteinaceous enterotoxins have been causing a significant number of food-poisoning outbreaks and illnesses. The European food safety authority (EFSA) reported that SEs accounted for 393 of the 843 documented outbreaks related to bacterial toxins in 2014 [1, 2]. A large variety of foods, such as meat and meat products, egg products, salads, bakery products and milk and dairy products including cheeses were affected [3], and symptoms following ingestion of nanogramme to microgram amounts include among others vomiting, diarrhoea, prostration, dizziness and abdominal pain [4, 5]. Meanwhile, 23 structurally related SE proteins with molecular masses of 22–28 kDa, documented stability to changing temperature and pH and resistivity to proteolytic digestion have been described in the literature [3, 6–8]. SEA is a single-chain 233 amino acid containing 27 kDa protein [9] and is the SE serotype most frequently involved in staphylococcal food poisoning (ca. 80 %) [10, 11]. Commission Regulation (EC) No 2073/2005 amended by Commission Regulation (EC) No 1441/2007 [12, 13] lays down so-called microbiological criteria for foodstuffs, which include enumeration of CPS colony forming units (CFUs) and detection of SEs for milk products, milk powders and whey powders. It specifies the number of replicate samples (*n* = 5), sample size (25 g) and the expected measurement result (“SEs not detected”) for such products to be placed on the market. Furthermore, it references the so-called European screening method (ESM) which has to be applied for analysis and which is based on toxin extraction, dialysis concentration and immunochemical detection. This screening method investigates the presence or absence of five SE sub-types (SEA-SEE) but cannot distinguish among them [14, 15].

Certified reference materials as one important pillar in quality assurance are valuable tools which assist laboratories in method validation and assessing the performance of the operated method. However, such materials are often not available. ANSES in its function as the European Union Reference Laboratory (EU RL) for CPS has highlighted the demand to have available suitable RMs for staphylococcal enterotoxins in food matrices. ANSES and JRC-IRMM agreed to collaborate to establish a reference material for SEA in cheese, currently seen as an important analyte/matrix combination. First priority was given to a material which supports the current legislation, which essentially means a material for presence/absence testing of SEA in cheese, using the above-mentioned ESM. In a recent publication, we reported on how to prepare suitable candidate reference materials on a large scale [16]. Moreover, homogeneity and short-term stability (dispatch conditions) have been evaluated and shown to be appropriate for the intended use of the materials [16].

This article describes the assessment of the long-term stability of these materials and the assignment of certified values to the materials by means of a laboratory intercomparison using the ESM with either of the two commercially available immunochemical detection steps. Moreover, an intra-laboratory assessment of the prescribed extraction and dialysis concentration step in the ESM was performed using a double-sandwich quantitative enzyme-linked immunosorbent assay (ELISA) to determine robust estimates of the recovery of SEA from the cheese matrix.

## Materials and methods

### Chemicals and consumables

Highly purified lyophilised SEA (purity >95 % as indicated on the product specification sheet) was obtained from Toxin Technology Inc. (Sarasota, USA). The lyophilised toxin provided was reconstituted with water following the instructions of the material provider, and further dilutions were made using the following buffer (PBS): 10 mM NaH_2_PO4 dodecahydrate, 145 mM NaCl, adjusted to pH 7.3 with HCl.

Sodium hydroxide 5 and 1 M, hydrochloric acid 5 and 1 M, as well as polyethylene glycol (PEG) 20000 were obtained from Merck, Darmstadt, Germany. Dialysis tubings (Spectra/Por® molecular porous regenerated cellulose membranes, MWCO 6000–8000) were obtained from Spectrum Laboratories Inc., Rancho Dominguez, CA, USA.

### Processing of reference materials, homogeneity assessment, short-term stability study

The candidate reference materials were processed as described elsewhere [16]. Homogeneity and short-term stability studies were performed as described previously [16] and revealed that the materials were sufficiently homogeneous for the intended use and can be shipped at ambient temperature without detectable loss of SEA in the cheese matrix.

### European screening method—sample preparation part

The full description of the ESM can be found elsewhere [17]. Briefly, 25.0 ± 0.1 g of samples are transferred into a glass beaker and 40 mL of osmosis or distilled water (38 ± 2 °C) are added. The mixture is homogenised by using an ultraturrax device, blender or stomacher. The sample is put on a horizontal shaker for at least 30 min. The sample is acidified by drop-wise addition of HCl to a pH of 3.5–4.0. The sample is centrifuged at 4 °C (15 min, 3130×*g*). The supernatant is checked for its pH; if the pH exceeds 4.5, HCl is added to achieve a pH of 3.5–4.0 and the sample is re-centrifuged. The sample is neutralised with NaOH to a pH of 7.4–7.6. A dialysis tube using a Spectra/Por membrane (MWCO 6–8 kDa) is prepared. The sample is poured into the dialysis tube by using a funnel with a glass wool filter for removal of suspended particles. The solution is dialysed overnight against 30 % (*w*/*v*) PEG solution at 5 ± 3 °C. The sample is re-suspended in PBS buffer, and the tube is rinsed extensively with the same buffer. All sample fractions are recovered in a glass vial. The final extract mass has to be in the range 5.0–5.5 g (5.0–5.8 g for sticky extracts, e.g. some types of cheeses).

### European screening method–analytical part and result interpretation

The commercially available immunoassays used for qualitative measurements were the VIDAS^®^ SET2 assay (bioMérieux, Marcy l’étoile, France) and the RIDASCREEN^®^ SET Total assay (R-Biopharm, Darmstadt, Germany). Those were applied for stability studies and the characterisation study, respectively. Thereby, the procedure outlined in the ESM had to be strictly followed. In the following, the ESM using the VIDAS^®^ SET2 assay is named ESM/VIDAS, and the ESM using the RIDASCREEN^®^ SET Total assay is named ESM/Ridascreen.

Both assays yield quantitative results. However, the results are not expressed in toxin mass fraction (e.g. ng toxin/g matrix), but either as an optical density (OD) in case of the Ridascreen assay or as a test value (TV) in case of the VIDAS assay. The ESM specifies a result at or above the so-called threshold (cutoff) value as SE types A-E detected in the test portions or referred to as “SEs present”. Likewise, a result below the threshold (cutoff) is classified as SE types A–E not detected in the test portions or referred to as “SEs absent”.

The test value (TV) of the sample is calculated by the automated VIDAS instrument as ratio of fluorescence value obtained for the unknown divided by fluorescence value obtained for the SEA-containing solution serving as so-called standard in the kit. The OD value is the direct read-out for the unknown sample using a microplate spectrophotometer. For the VIDAS detection step, the threshold is fixed at a test value of 0.13 (established during assay validation at the kit provider). For the Ridascreen detection step, the cutoff value is calculated by adding 0.15 absorbance units (AUs) to the OD value obtained for the negative control included in the kit, as prescribed by the kit provider. The performance and effectiveness of the ESM has been demonstrated through a number of intra- and interlaboratory validation studies targeting SEA, SEC, SED and SEE in several foodstuffs [14, 15].

### Long-term stability studies

Long-term stability studies for evaluating suitable storage conditions were conducted as isochronous stability studies [18] and were carried out for the two SEA-containing materials (IRMM-359b, IRMM-359c).

For the study running over 1 year, two units were selected per time/temperature point, and from each unit, three sub-samples were analysed. Storage temperatures were 4 and −20 °C, and the reference temperature was set to −70 °C. The storage times were 0, 4, 8 and 12 months. After the indicated storage periods, samples were transferred to storage at −70 °C until analysis. Measurements had to be split over five days to perform the 42 analyses. The ESM/VIDAS was used for analysis. Data were checked for outliers using the Grubbs test (99 % confidence level), and linear regression analysis as a function of time was performed. Slopes were tested for significance using a *t* test at the 95 % confidence level.

In addition, a 2-year study was performed, for temperatures 4 and −20 °C and a reference temperature of −70 °C. Seen the excellent results for the 1 year stability study, only samples with storage times of 0 and 24 months were finally analysed. Two units per storage time and temperature were selected, and three replicate analyses were performed from each unit using the ESM/VIDAS as described above. Measurements had to be split over 3 days to perform the analysis. Data were evaluated as described above.

### Intra-laboratory study for assessing SEA recovery

A study was conducted to assess the recovery of SEA from the cheese matrix. To this end, the sample preparation procedure described above was followed (extraction with subsequent dialysis concentration), and a double-sandwich ELISA (see below) was used for SEA quantification. IRMM-359a (blank) samples were spiked at 0.1 and 0.25 ng/g, respectively, before analysis, and IRMM-359b (nominal concentration 0.1 ng SEA/g cheese) and IRMM-359c (nominal concentration 0.25 ng SEA/g cheese) were analysed as is. Pure SEA (Toxin Technology, Sarasota, USA), nominally 1 mg/vial was reconstituted in double-distilled water and gravimetrically diluted 1:10 in PBS buffer. The SEA concentration in the solution used for spiking was analysed using an amino acid analysis (AAA) method based on liquid chromatography—isotope dilution—tandem mass spectrometry (LC-ID-MS/MS) [19]; the result confirmed the nominal value within the uncertainty of the measurement method. The recovery was therefore defined as found value with the quantitative ELISA (spiked materials or CRMs) divided by the respective nominal values (spiked material or CRM, see above). In order to establish within-laboratory reproducibility conditions, the work for each sample type (two spiked samples, two candidate CRM samples) was split over three to five days, and the work was assigned in random order to one of three analysts. In total, 24 independent sample replicates from each of the blank materials spiked with SEA at 0.1 and 0.25 ng/g were analysed, and six independent sample replicates from each candidate CRM (IRMM-359b, nominal concentration 0.1 ng SEA/g cheese, and IRMM-359c, nominal concentration 0.25 ng SEA/g cheese) were analysed.

### Quantitative SEA ELISA

Extracts prepared in the frame of the recovery study were analysed using a double-sandwich quantitative ELISA developed at ANSES [20] and further optimised using commercially available antibodies (Toxin Technology, Sarasota, FL, USA), namely affinity-purified sheep anti-SEA IgG (product code SLAI101) as capture antibodies and affinity-purified rabbit anti-SEA IgG (product code LAI101) as primary detection antibodies, respectively. Goat anti-rabbit IgG coupled to HRP was used as secondary detection antibody (KPL, product code 074-1516). Each prepared extract was analysed in duplicate undiluted and/or appropriately diluted in PBS buffer containing 0.2 % (*w*/*v*) gelatine and 0.1 % (*w*/*v*) Tween-20 so as to fall within the calibration range. The presence of toxins was revealed by adding the substrate ABTS and spectrophotometric measurement at 405/630 nm after colour development. A calibration curve was prepared with dilutions from SEA working solution with five concentration levels between 0 and 0.5 ng SEA/mL (duplicate calibration points per level). Positive and negative control samples (milk extracts spiked at 0.275 ng SEA/mL or unspiked) were co-analysed to prove the validity of the method. The LOQ was estimated as 0.05 ng SEA/mL from a total of 20 measurements of a buffer blank obtained over a period of 5 months by various analysts; this corresponds to 0.01 ng/SEA extract (test portion of 25 g converted into 5 mL of extract [17]).

### Interlaboratory comparison to acquire data for reference material certification

A laboratory intercomparison was organised. Laboratories were selected based on their technical competence and experience to apply the ESM. Having the method operated under ISO/IEC 17025 accreditation was not mandatory, but considered as an asset. In total, 13 laboratories participated in the characterisation study: six laboratories used the ESM/VIDAS, five laboratories used the ESM/Ridascreen and two laboratories applied both ESM/VIDAS and ESM/Ridascreen. For those two laboratories, it shall be noted that independent samples were prepared for analysis using either detection step.

Each laboratory received three sets of IRMM-359 (one set consists of one sachet of IRMM-359a (blank), one sachet of IRMM-359b (very low level SEA) and one sachet of IRMM-359c (low level SEA). The sets were selected randomly over the whole candidate CRM batch. Within a day, three independent sub-samples of each sachet had to be prepared and analysed, and this work was repeated on two more days, thus amounting to nine analyses per material in total. Laboratories had to follow the technical specifications that were provided together with the samples. In particular, the reconstitution of the cheese powder had to be performed as follows: 9.9 g of distilled water was to be added to 15.1 g powder. The sample was then to be homogenised by adding a magnetic stirring bar to each powder/water mixture and stirred for 10–15 min at room temperature. Thereafter, the protocol stipulated in the ESM had to be strictly followed. Special emphasis during the technical scrutiny of the acquired data was attributed to the accuracy of reconstitution (prescribed masses), pH adjustments during extract preparation to respect the specified intervals defined in the ESM, and final extract mass prescribed in the ESM (5.0–5.8 g for sticky matrices such as cheese). Laboratories had to report both the obtained test values (VIDAS) and/or OD values (Ridascreen) together with the conclusions SEA detection (“presence”) or SEA non-detection (“absence”).

## Results and discussion

### Processing of the reference materials, homogeneity and short-term stability assessment

Processing of the candidate reference materials (IRMM-359a, blank cheese powder, IRMM-359b, cheese powder containing SEA at a nominal concentration of 0.1 ng/g cheese, and IRMM-359c, cheese powder containing SEA at a nominal concentration of 0.25 ng/g cheese), assessment of homogeneity and stability during dispatch is described elsewhere [16]. Homogeneity studies for materials b and c indicated that the homogeneity of the materials is fit for their intended use. A 4-week short-term stability demonstrated that the materials can be shipped to the customer at ambient temperature [16].

### Stability studies

#### One-year stability study

The data were evaluated individually for each temperature. The results were screened for outliers using the single and double Grubbs tests at a confidence level of 99 %. For SEA in IRMM-359b, one outlier was found (4 °C, 12 months, value 1.00). As no technical reason for this outlier could be found, the result was retained for statistical analysis. For SEA in IRMM-359c, one outlier was found and excluded from further data evaluation for technical reasons (part of sample lost during extraction). The data were evaluated against storage time and regression lines of test values versus time were calculated (Electronic supplementary material Fig. S1). The slopes of the regression lines were tested for statistical significance. For SEA in IRMM-359b, a statistically significant trend was obtained at 4 °C (95 % confidence level). However, this trend can be regarded as technically irrelevant, as it is caused by a statistical outlier (see above). At −20 °C, the slope of the regression line was not significantly different from zero (95 % confidence level). For SEA in IRMM-359c, the slopes of the regression lines were not significantly different from zero (95 % confidence level) at both 4 °C and −20 °C. The material can therefore be stored at −20 °C.

#### Two-year stability study

An isochronous study was scheduled over a period of 2 years. However, seen the excellent results of study over 1 year (suitable material stability at 4 and −20 °C), it was decided to only measure at time points 0 and 24 months. The data were evaluated as described for the one-year study. No outlier for SEA in IRMM-359b and IRMM-359c was found. The slopes of the regression lines were tested for statistical significance (Electronic supplementary material Fig. S2). For SEA in IRMM-359b and IRMM-359c, the slopes of the regression lines were not significantly different from zero (95 % confidence level) at both 4 °C and −20 °C. It was confirmed that the material can be stored at −20 °C.

The stability of the materials will be regularly monitored throughout their lifetime (time span from material release to material being sold out) in the frame of IRMMs regular CRM stability monitoring programme.

#### Intra-laboratory study to assess the recovery of SEA in the cheese matrix

**Fig. 1:**
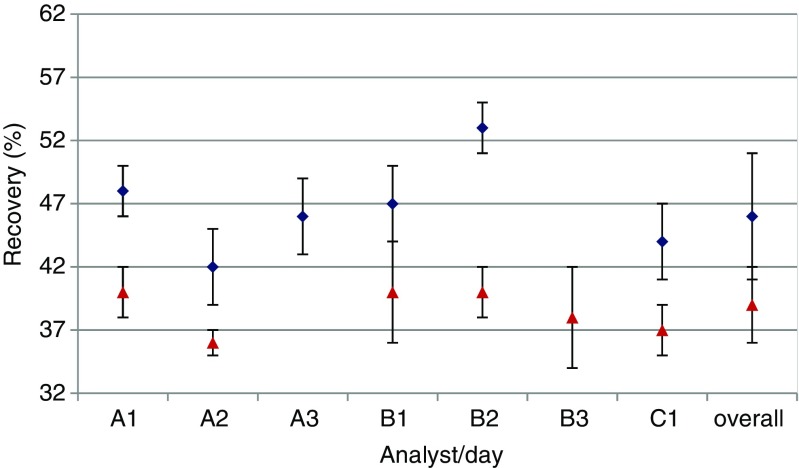
Blank cheese samples spiked with SEA at 0.1 ng/g (*blue diamonds*) and 0.25 ng/g (*red triangles*). Performance of analysts (*A–C*) over time (measurement days 1–3) and overall performances. The *error bars* represent the variability of the four independent analyses per analyst and day (24 independent data in case of the overall values)

**Fig. 2:**
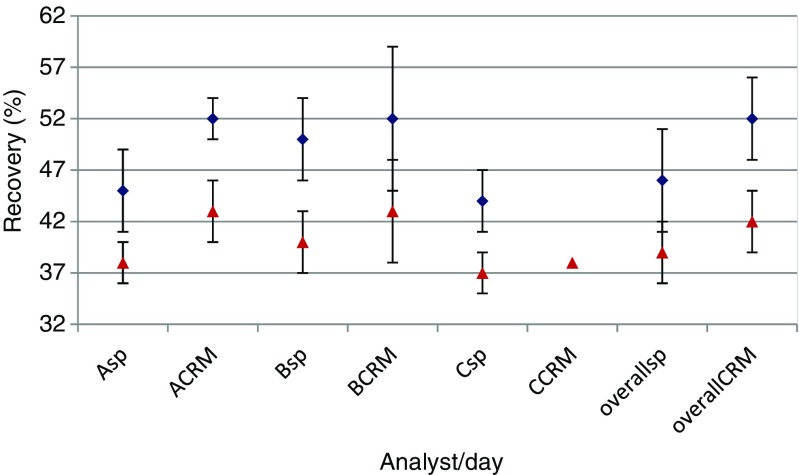
Comparison of blank cheese samples spiked before analyses (*sp*) and CRM samples having nominally the same SEA level (*CRM*); 0.1 ng/g level (*blue diamonds*) and 0.25 ng/g level (*red triangles*). The *error bars* give the overall analyst performance (overall performance) over 3 days

The aim of the study was to provide a robust estimation of toxin recovery from the cheese matrix when using the ESM and the sensitive quantitative ELISA (LOD 0.015 ng/mL as determined in an in-house validation study). The results are summarised in Figs. 1 and 2. Seen the tedious sample preparation procedure (manual workload per sample), only a limited amount of analyses was performed (*n* = 52). However, the study was designed in a way to allow a sound estimation of an achievable recovery of SEA from the cheese matrix and to study comparability of results from freshly spiked samples versus CRMs (different analysts, different days, random allocation of samples to an analyst etc.).

Figure 1 shows the comparison between different analysts (A, B, C) over time (days 1, 2, 3) when spiking blank cheese powder with SEA at low (blue) or high (red) level. It was randomly decided which analyst would analyse four independent samples per day on at least 1 to maximum 3 days. Generally, it can be concluded that recoveries were somewhat lower at the high spiking level; however, taking into account the overall values (all analysts, all days), no significant difference in the mean recovery of about 45 % can be found between the two spiking levels (46 ± 5 % for the low level, 39 ± 3 % for the high level). The obtained values can therefore be seen as a robust estimate of obtainable recovery using the ESM and that specific cheese matrix.

Figure 2 shows the comparison between mean values obtained for spiked samples and CRM samples by each analyst and an overall comparison, again for both spiking levels/CRMs (blue/red). Again, recovery values were somewhat lower for IRMM-359c compared to IRMM-359b; however, there’s no indication of a significant difference, especially when comparing the overall data. Secondly, when comparing mean values produced by analyst when analysing CRM and spiked samples for a given SEA level, an overlap of datasets is obtained, indicating equivalence of results among samples spiked before analysis and CRM samples. In all cases however, the obtained recovery values for CRM samples were somewhat higher than those of the spiked samples (52 ± 4 % for IRMM-359b (low level), 42 ± 3 % for IRMM-359c (high level)).

To further elaborate on the obtained results, extracts were prepared from blank cheese powder and spiked post-extraction with SEA at the 0.1 ng/g level (*n* = 6) and the 0.25 ng/g level (*n* = 6). The obtained recoveries of 97 ± 3 % (interval 94–102 %) for the lower level and 86 ± 10 % (interval 72–101 %) for the higher level indicate that the loss of SEA is not primarily caused by matrix effects in the extract. The losses rather occur to a substantial degree in the sampling extraction part of the procedure. Most likely, the last working steps, i.e. rinsing of membrane to remove traces of polyethylene glycol, addition of buffer, solubilisation of toxin, and essentially the quantitative transfer of the ca. 5 mL extract to another container are contributing to obtaining a low recovery. Technically speaking, the most probably explanation is that toxin sticks to the inside of the membrane and cannot be recovered quantitatively.

Seen that comparable recoveries in spiked and CRM samples were obtained for each level, it can be concluded that SEA detection and quantification was not impaired in the CRM samples (preparation process such as milling of material and especially lyophilisation did not have a negative impact). Secondly, a comparable recovery can be obtained when different analysts analyse different samples from the same batch over time. Finally, it shall be emphasised that the shown variability levels in the study represent either repeatability (error bars in Fig. 1) or within-laboratory reproducibility (error bars in Fig 2). However, the measurements uncertainty has not yet been established for this method, and several factors contributing to the measurement uncertainty such as calibrant purity and uncertainty from value assignment, dilution and sample manipulations of the spiked or reference material, etc. are not yet taken into account. In essence, the measurement uncertainty would be larger than the shown variability values.

The mean SEA recovery of around 45 % as obtained in this study compares well with work published earlier [21], where SEA recovery in soft cheese at spiking levels of 0.3 and 0.7 ng/g was found to be 50 and 47 %, respectively. The results of the intra-laboratory study presented here contribute to better assess the method capabilities of the ESM and can be used by laboratories as a benchmark of what experienced analysts can achieve with the ESM. It shall be noted that the result is matrix-dependent and also depends on the capability of the analyst to operate the method accurately. A higher recovery might possibly be obtained with another sample preparation procedure, avoiding the rather tedious PEG dialysis concentration. However, as the ESM has the status of a legally recognised reference method, a good assessment of its performance is essential and important, last but not least for laboratories applying the ESM to better understand comparability of their results with those obtained by others, for instance in respective PT rounds.

#### Characterisation study and value assignment

Fifteen datasets (eight ESM/VIDAS and seven ESM/Ridascreen) were received and first checked for compliance with the requested analysis protocol and for their validity based on technical reasons. The criteria considered during the evaluation were the adherence to the prescribed masses of cheese powder and water to be used for reconstitution, the pH value after acidification of the sample to be in the interval 3.5–4.0; the pH value after centrifugation to be <4.5 in the supernatant; the pH value after neutralisation to be in the interval 7.4–7.6, and the final mass of the sample to be in the interval 5.0–5.8 g. Table 1 lists the individual results that had to be discarded due to non-compliance with the prescribed procedure.

**Table 1:** Datasets that showed non-compliances with the analysis protocol and technical specifications, with consequence that data were not considered for establishing the certified value

Material	Lab code	Number of replicates	Reason for rejection
IRMM-359a	B	4	Reconstitution masses outside range
	C	8	pH after neutralisation outside range
	G	3	Final extract mass outside range
	K	8	pH after acidification outside range
IRMM-359b	B	4	Reconstitution masses outside range
	B	2	Final extract mass outside range
	K	7	pH after acidification outside range
IRMM-359c	B	1	Reconstitution masses outside range
	G	1	Final extract mass outside range
	J	1	Partial sample loss during manipulation
	K	7	pH after acidification outside range

Both assays deliver quantity values (so-called test values for the VIDAS assay; optical density values for the Ridascreen assay, see above). However, the results of analysis are finally expressed as SEs A-E not detected (“negative”, “absent”) or SEs A-E detected (“positive”, “present”), which is accomplished by comparison of the obtained value with the respective cutoff (threshold) of the assay (the discriminator between presence and absence, in practice a limit of detection). A summary of the obtained raw data (test values; OD values) is presented in Table 2.

**Table 2:** Summary of direct read-out results as obtained in the characterisation study

Material	Mean value^a^	Interval^b^
ESM/VIDAS SET2		
IRMM-359a	0.01	0.00–0.05
IRMM-359b	1.14	0.47–1.53
IRMM-359c	1.97	1.10–2.42
ESM/Ridascreen SET Total		
IRMM-359a	0.08	0.01–0.19
IRMM-359b	0.61	0.28–1.11
IRMM-359c	1.36	0.45–2.31

The values in the table are test values (VIDAS SET2) or absorbance units (Ridascreen SET Total) as defined in the European screening method [17]

^a^Mean of mean of eight datasets (ESM/VIDAS) and seven datasets (ESM/Ridascreen)

^b^Interval (lowest and highest individual value)

It shall be noted that these values are meant as information values for the laboratories when comparing the results they obtain with the results of the assays used for the characterisation of the reference materials. For establishing the certified values, however, these values were converted into presence/absence classifications as outlined above.

The following results were thus obtained: IRMM-359a, 112 valid results, all classified as “SEs detected”; IRMM-359b, 122 valid results, all classified as SEs detected; IRMM-359c, 125 valid results, all classified as SEs detected. Based on this, the certified values as expressed as diagnostic specificity (blank material) or diagnostic sensitivity (SEA-containing materials), as defined in the formulae below and elsewhere [22, 23]:

$$ \begin{array}{cc}\hfill \mathrm{Specificity}=\frac{\mathrm{TN}}{\mathrm{TN}+\mathrm{F}\mathrm{P}}\times 100\hfill & \hfill \mathrm{Sensitivity}=\frac{\mathrm{TP}}{\mathrm{TP}+\mathrm{F}\mathrm{N}}\times 100\hfill \end{array} $$

with TN as true negative, TP as true positive, FN as false negative and FP as false positive. TN and TP refer to correct classification of a given sample (positive result for a sample that is positive, negative result for a sample that is negative), whereas FN and FP refer to incorrect test results for a given sample (negative result for a sample that is positive, positive result for a sample that is negative).

The certified value has the meaning of a detection probability. Diagnostic sensitivity and specificity are widely used in food and clinical microbiology [24, 25] to describe performance characteristics of assays applied in these fields, but in this specific context, they are used as a means to quantitatively describe the presence/absence of the analyte (SEA) in the matrix, based on the results obtained.

Table 3 shows the certified values that were established for the presence or absence of SEA in the materials IRMM-359a-c. The meaning of the certified values can be summarised as follows:

**Table 3:** Certified values for the three cheese materials

Material	Technically valid individual results (*n*)	Specificity (%)^a, b^	Sensitivity (%)^a, b^	One-sided lower confidence limit (%)^c^
IRMM-359a	112	100	–	97.3
IRMM-359b	122	–	100	97.5
IRMM-359c	125	–	100	97.6

The presence/absence certification (see text for explanation and details) is expressed in either diagnostic specificity (blank material) or diagnostic sensitivity (SEA-containing materials)

^a^As defined in the formula depicted in the text and in [18]

^b^As determined using the European screening method (ESM) with the VIDAS SET2 detection step and the Ridascreen SET Total detection step. The certified value is based on eight accepted datasets of the ESM with the VIDAS SET2 detection step and seven accepted datasets of the ESM with the Ridascreen SET Total detection step. The certified value is traceable to the SI

^c^The lower confidence limit is based on the results of 15 laboratories. It is determined assuming a Poisson distribution [26] with *n* technically valid (correct) and 0 incorrect results. The value holds for a 95 % level of confidence

IRMM-359a—using the ESM and IRMM-359a, the probability of obtaining a correct result was 100 %, with a confidence interval ranging from 97.3 to 100 % at the 95 % confidence level (*α* = 0.05; *n* = 112). IRMM-359b—using the ESM and IRMM-359b, the probability of obtaining a correct result is 100 %, with a confidence interval ranging from 97.5 to 100 % at the 95 % confidence level (*α* = 0.05; *n* = 122). IRMM-359c—using the ESM and IRMM-359c, the probability of obtaining a correct result is 100 %, with a confidence interval ranging from 97.6 to 100 % at the 95 % confidence level (*α* = 0.05; *n* = 125).

### Metrological traceability of the certified values

The measurement results for classifying the material in terms of presence or absence of SEA were generated by adhering to the ESM [17]. The sample preparation protocol for extraction and dialysis concentration as well as the analytical detection step with a commercial assay had to be strictly followed. Therefore, the measured properties are so-called operationally defined. The identity of SEA was assessed by SDS-PAGE (molecular mass deduced from gel) and confirmatory ELISA (SEA-specific). Metrological traceability of the obtained results is based on the traceability of all relevant input factors. Instruments in individual laboratories were verified and calibrated with tools ensuring traceability to the International System of Units (SI). Consistency of results in the interaboratory comparison demonstrates that all relevant input factors were covered. As the assigned values are combinations of agreeing results individually traceable to the SI, the assigned values themselves are traceable to the SI as well.

## Conclusions

One blank and two SEA-containing cheese materials could be successfully certified for testing of the presence/absence of SEA. The certified values, expressed as either diagnostic specificity (blank) or diagnostic sensitivity (SEA-containing materials), are traceable to the International System of units (SI). The materials are intended to be used for method performance control and validation purposes. A laboratory using these CRMs for analyses compares the result it generates with the respective certified value (absence of SEA in IRMM-359a, presence of SEA in IRMM-359b and IRMM-359c). Furthermore, the laboratory can compare the quantitative values obtained from the ESM/VIDAS and the ESM/Ridascreen (test value and OD, respectively) with those listed as additional material information on the certificates of the three materials. The useful information about SEA recovery in the cheese matrix will furthermore provide laboratories with a performance benchmark and will assist in correct implementation of the method or can serve for method performance assessment.

## Electronic supplementary material

Below is the link to the electronic supplementary material.

ESM 1 (PDF 62 kb)
