# Supplementary material for: Certified reference materials for testing of the presence/absence of Staphylococcus aureus enterotoxin A (SEA) in cheese
Source: Anal Bioanal Chem. 2016 May 24;408:5457–65. doi: 10.1007/s00216-016-9642-5 (PMC4939153; doi:10.1007/s00216-016-9642-5)
Supplement: Supplementary file 1 — (PDF 62 kb) [file 216_2016_9642_MOESM1_ESM.pdf]

Analytical and Bioanalytical Chemistry

Electronic Supplementary Material

Certified reference materials for testing of the presence/absence of *Staphylococcus aureus* enterotoxin A (SEA) in cheese

R. Zeleny, Y. Nia, H. Schimmel, I. Mutel, J.-A. Hennekinne, H. Emteborg, J. Charoud-Got, F. Auvray

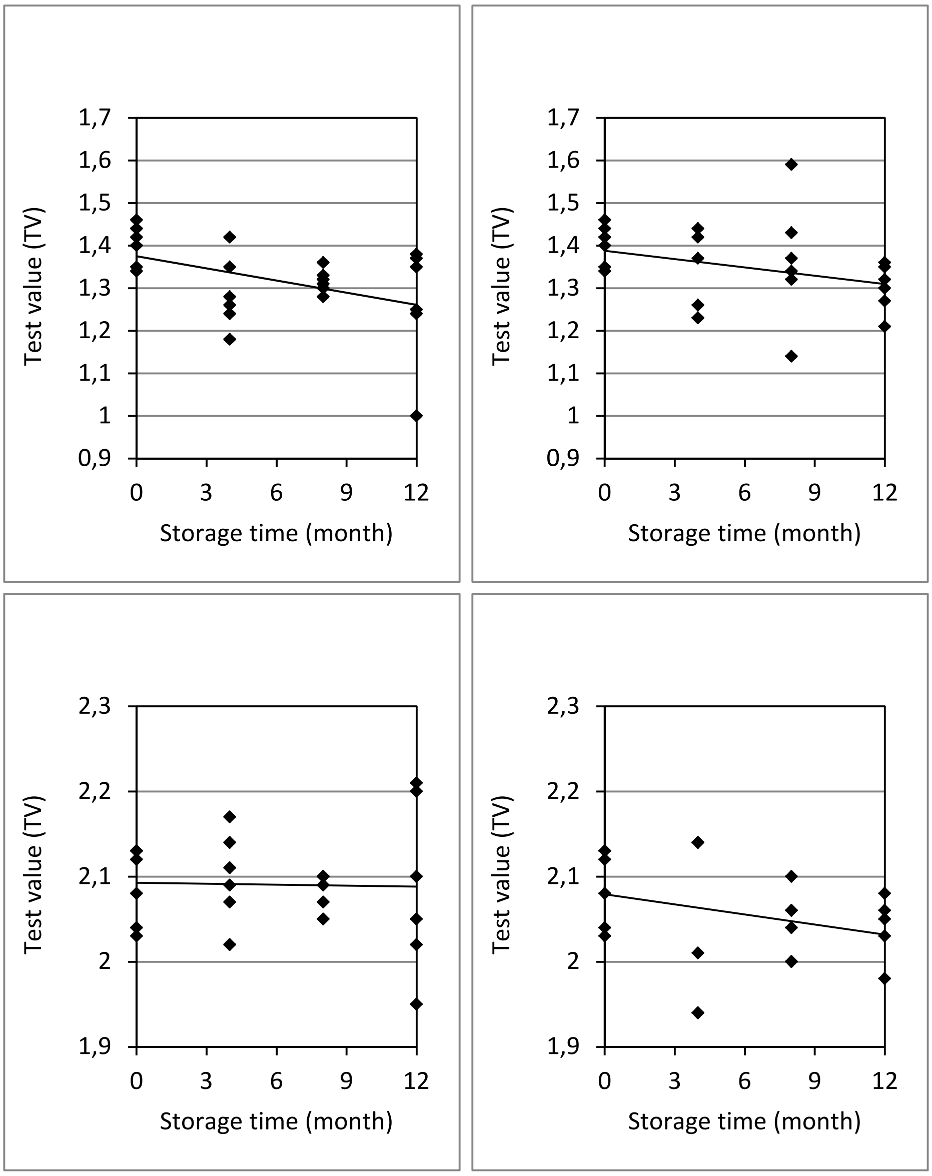

Fig. S1. Results of the one-year stability study of IRMM-359b and IRMM-359c using the ESM/VIDAS for analysis. The obtained results per individual time point and the respective regression lines are shown. Upper part: IRMM-359b, storage temperature 4^{0}C (left) and -20 ^{0}C (right). Lower part: IRMM-359c, storage temperature 4^{0}C (left) and -20 ^{0}C (right)

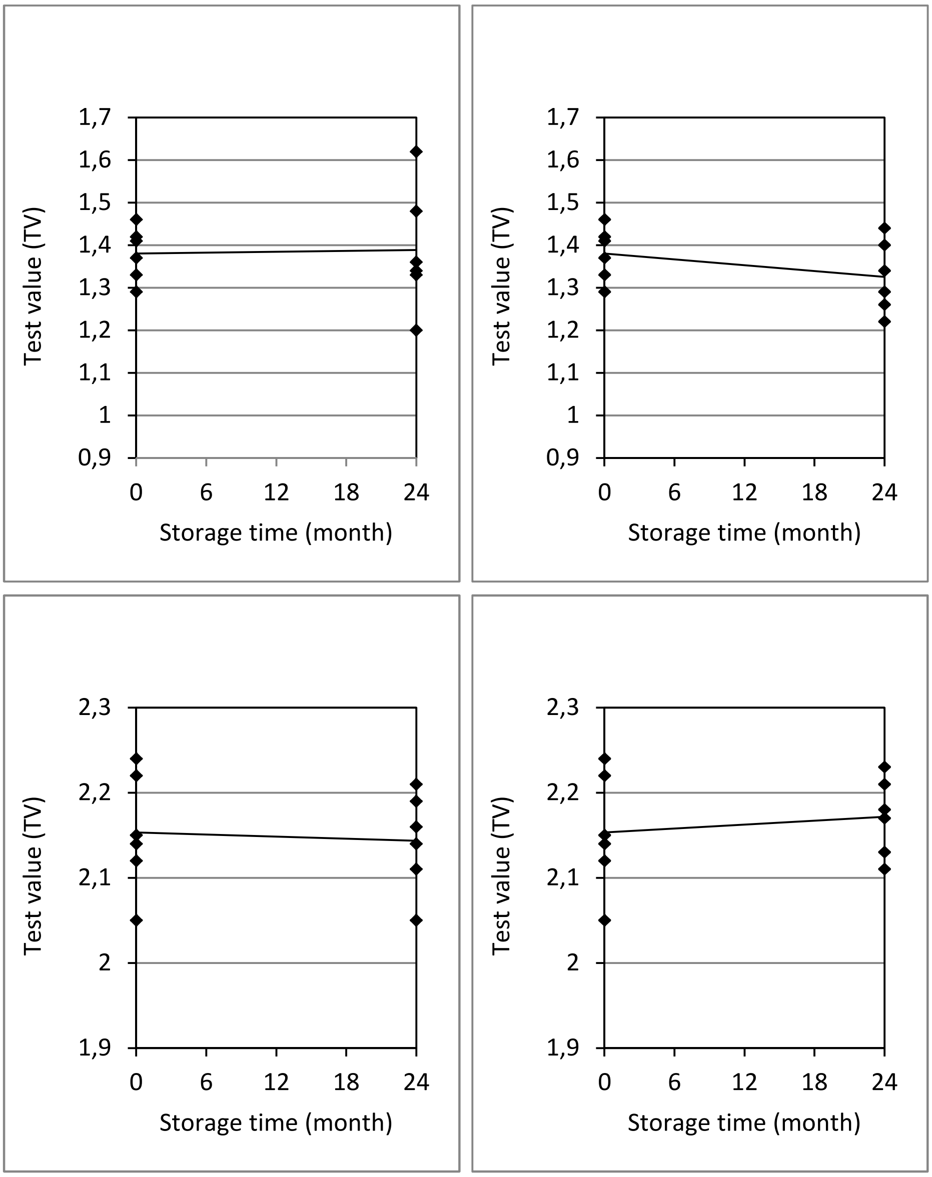

Fig. S2. Results of the 2-year stability study of IRMM-359b and IRMM-359c using the ESM/VIDAS for analysis. The obtained results per individual time point and the respective regression line are shown. Upper part: IRMM-359b, storage temperature 4^{0}C (left) and -20 ^{0}C (right). Lower part: IRMM-359c, storage temperature 4^{0}C (left) and -20 ^{0}C (right)
